# Frailty and Exercise Training: How to Provide Best Care after Cardiac Surgery or Intervention for Elder Patients with Valvular Heart Disease

**DOI:** 10.1155/2018/9849475

**Published:** 2018-09-13

**Authors:** Egle Tamuleviciute-Prasciene, Kristina Drulyte, Greta Jurenaite, Raimondas Kubilius, Birna Bjarnason-Wehrens

**Affiliations:** ^1^Rehabilitation Department, Lithuanian University of Health Sciences, Eiveniu g. 2, LT-50161 Kaunas, Lithuania; ^2^Faculty of Medicine, Lithuanian University of Health Sciences, A. Mickevičiaus g. 9, LT-44307 Kaunas, Lithuania; ^3^Institute of Cardiology and Sports Medicine, Department Preventive and Rehabilitative Sport and Exercise Medicine, German Sport University Cologne, Am Sportpark Muengersdorf 6, 50933 Cologne, Germany

## Abstract

The aim of this literature review was to evaluate existing evidence on exercise-based cardiac rehabilitation (CR) as a treatment option for elderly frail patients with valvular heart disease (VHD).* Pubmed* database was searched for articles between 1980 and January 2018. From 2623 articles screened, 61 on frailty and VHD and 12 on exercise-based training for patients with VHD were included in the analysis. We studied and described frailty assessment in this patient population. Studies reporting results of exercise training in patients after surgical/interventional VHD treatment were analyzed regarding contents and outcomes. The tools for frailty assessment included fried phenotype frailty index and its modifications, multidimensional geriatric assessment, clinical frailty scale, 5-meter walking test, serum albumin levels, and Katz index of activities of daily living. Frailty assessment in CR settings should be based on functional, objective tests and should have similar components as tools for risk assessment (mobility, muscle mass and strength, independence in daily living, cognitive functions, nutrition, and anxiety and depression evaluation). Participating in comprehensive exercise-based CR could improve short- and long-term outcomes (better quality of life, physical and functional capacity) in frail VHD patients. Such CR program should be led by cardiologist, and its content should include (1) exercise training (endurance and strength training to improve muscle mass, strength, balance, and coordination), (2) nutrition counseling, (3) occupational therapy (to improve independency and cognitive function), (4) psychological counseling to ensure psychosocial health, and (5) social worker counseling (to improve independency). Comprehensive CR could help to prevent, restore, and reduce the severity of frailty as well as to improve outcomes for frail VHD patients after surgery or intervention.

## 1. Introduction

Worldwide, the population is aging, and the associated challenges for the healthcare system are significant. There is a clear association between degenerative valve disease, older age, and increasing life expectancy [1, 2]. According to the Euro Heart Survey on Valvular Heart Disease (VHD), patients with diagnosed VHD are often older, with a higher prevalence of other cardiovascular risk factors and comorbidities [2].

Careful monitoring, adequate medication, and perfect surgery or intervention timing is key to a successful treatment of VHD. Due to the shift in patient population, the profile of patients who are referred to cardiac surgeon changed dramatically. The share of surgically treated elderly patients (≥80 years old) increased from 2.6-7% to 9.3-12% in a period of 10 years [3, 4]. Innovation in cardiac surgery techniques [5], advance in anesthesiology, and early postsurgery care led to a lower risk of cardiac surgery in elderly patients [4] and evidence of improved outcomes with valve surgery or transcatheter aortic valve implantation (TAVI) [6, 7]. However, older patients still have higher mortality and morbidity rates, more frequent complications related to surgery, and longer stay in hospital compared to younger patients [3, 4, 6].

Disease complexity in the elderly raises questions about other risk factors that exist upon well-known and established prognostic factors. Frailty is a common and relevant geriatric syndrome that could be defined as a biological syndrome with reduced reserve and resistance to stressors, resulting from cumulative deficits across multiple physiologic systems and causing vulnerability to adverse outcomes [8]. Most of the studies analyzed frailty before surgery/intervention as prognostic tool for later outcomes [9] and frailty is a reliable prognostic factor for mortality, morbidity, major complications [10–16], functional decline [17], quality of life [18], and risk of delirium after procedure [19, 20]. Frailty status can be changed and its assessment should not only be for prognosis but also lead to “pre-rehabilitation” interventions [21] and clear comprehensive cardiac rehabilitation (CR) recommendations [9, 22]. Physical rehabilitation for frail older people can positively affect physical fitness and this effect may be related to level of frailty in a setting of long-term care [23]. On the other hand, there are no clear recommendations concerning what methods and tools are sensitive enough and could be used to detect existence* and* dynamics of frailty syndrome in this particular patient population.

The aim of this literature review was to evaluate existing evidence on exercise-based cardiac rehabilitation (CR) as a treatment option for elderly frail VHD patients. This literature review aimed to assess the following issues:

1. Are assessment tools used for frailty screening sensitive enough to show improvement in frailty status after exercise-based CR?
2. What exercise interventions would be the most beneficial for frail patients?
3. Would the change of frailty status improve outcomes on levels of disability, functional capacity, and quality of life after surgical/interventional VHD treatment beyond the effects of the surgery/intervention itself?

## 2. Methods

We conducted a literature review of articles published from 1980 to January 2018. Literature search was performed using the PubMed database. Relevant studies were identified using the following key words: valve surgery/TAVI and exercise training, cardiac rehabilitation, moderate aerobic exercise training, high intensity interval training, resistance training, strength training, exercise capacity, prognosis, mortality, frailty, and sarcopenia. Additionally, we manually searched the bibliographies of all included articles.

**Figure 1:**
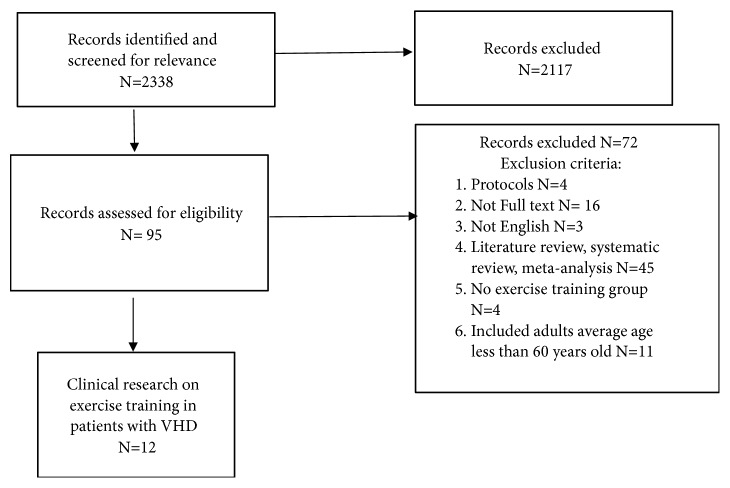
Flowchart of the selection of publications included in literature review related to valve surgery and exercise training.

**Figure 2:**
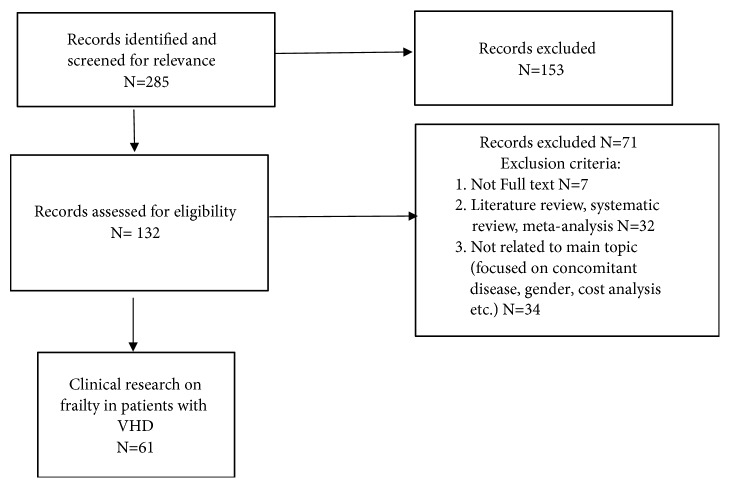
Flowchart of the selection of publications included in literature review related to valve surgery and frailty.

The search was limited to publications related to adults over 60 years old and published in English (see Figures 1 and 2).

## 3. Results

### 3.1. Are Assessment Tools Used for Frailty Screening Sensitive Enough to Show Improvement in Frailty Status after Exercise-Based CR?

In order to answer first research question we describe most often used frailty tools for screening in patients with VHD and compare results with frailty assessment methods used in a setting of CR.

Large variety of methods and instruments are used to describe existence or/and level of frailty for patients with VHD (Table 1). Most of the conducted studies were designed to evaluate various outcomes after surgery or intervention. The results of studies evaluating prevalence of the frailty and frailty status strongly depended upon used assessment tools and variables measured [24]. Prevalence of frailty varied from 20-26% to 68-82%, according to the scale and the population examined [9, 24]. A systematic review and meta-analysis by Anand et al. evaluated the relationship between preoperative frailty and outcomes following TAVI and demonstrated that the proportion of frail patients varied greatly across the different studies from 5 to 83% [25]. Although frailty has a good predictive value, results of the different studies are inconclusive and/or contradictory even with the same tools and similar populations. Also there is a gap of evidence concerning frailty tools sensitivity in this particular patient population. The most frequently used frailty assessment procedures for patients with VHD are discussed below.

#### 3.1.1. Fried Phenotype Frailty Index Assessment (FFS)

FFS represents the definition of physical frailty and it is based on the results of the Cardiovascular Health Study and the Women's Health and Aging Studies [8]. It has been widely adopted and consists of five health domains: nutrition (unintentional weight loss), physical exhaustion (CES–D (depression) scale), low energy expenditure (or inactivity status) (Minnesota Leisure Time Activity questionnaire), mobility (5-meter walking test (5MWT)), and muscular strength (dominant hand handgrip strength) [8]. FFS covers all domains of physical frailty and is recommended by the American Geriatrics Society [26]. FFS scale also could be named as frailty index from Cardiovascular Health Study (5-item CHS).

However, the study that validated the FFS was performed with general population. There is no information about VHD patients, and the share of very old individuals included in the study was relatively low (3.6% of patients were 85 years or older) [8]. Moreover, used methods were subjective and based on qualitative manner and could not precisely evaluate cognitive function and level of independence. Enhanced FFS (or 7-item expanded CHS) with additional testing of cognitive function (using Mini Mental State Examination (MMSE)) and depressed mood was implemented [24, 27]. Another example of FFS modification (modified fried frailty criteria) was scale for frailty evaluation that includes similar domains by using different methods to describe malnutrition (hypoalbuminemia) and clearly describe level of independence with Katz index [13, 28–30].

FFS [12, 24, 31–35] and scale modifications are widely used in studies involving VHD patients for frailty screening and unfavorable intervention/surgery outcome prediction. FFS was an accurate frailty assessment tool for prediction of increased hospitalization costs after cardiac surgery [31]. Some authors did not find any relation between frailty (measured by FFS) and mortality, morbidity, or increased length of stay in the hospital [33, 36, 37]. However the studies concluded that frail patients are more often referred to cardiac rehabilitation facilities [33, 36]. In a large study from Arnold et al. (N=2830) the authors demonstrated that frailty measured by FFS was a good predictor for negative short-term outcome (mortality, low quality of life) [32]. Similar results were reported in other studies [34, 35, 38, 39]. Forcillo et al. included high- and extreme-risk patients undergoing TAVI and found that serum albumin, Katz index, and 5MWT were associated with increased risk of adverse outcomes, but only albumin was predictive of 30-day all-cause mortality [28]. In contrast, in two studies from Afilalo et al. (2012 and 2017) the FFS and its modifications were inferior compared to other assessment tools (5MWT alone and EFT) for frailty screening [12, 24]. We failed to find any study that would be designed to evaluate FFS sensitivity for patients with VHD or in which FFS would be used in cardiac rehabilitation settings. According to our analysis, although FFS is a traditional method for frailty syndrome diagnosis and screening, it may be not reasonable for frailty dynamics after comprehensive CR program.

#### 3.1.2. Multidimensional Geriatric Assessment (MGA) or Modified Geriatric Baseline Examination (MGBE)

Stortecky et al. and Schoenenberger et al. showed MGA/MGBE to be a good predictor of mortality and major adverse cardiovascular and cerebral events (MACCE) [14] as well as functional decline or death [17] after TAVI. This tool describes frailty by results of MMSE, Mini Nutritional Assessment (MNA), Katz index, independence in instrumental activities in daily living (IADL), Time Up and Go (TUG), and a subjective mobility disability (defined as a decreased frequency of walking 200 m and/or of climbing stairs). This tool was used in several studies and resulted in good predictive value [14, 17, 19]. It has all necessary elements for frailty assessment: mobility (TUG and subjective mobility evaluation), nutrition (MNA), cognitive functions (MMSE), and independence (ADL, IADL).

In addition, Eichler et al. showed that this instrument could be used in CR settings, although there were no changes in Katz index and IADL results after 3-4 weeks of inpatient CR for patients after TAVI [40]. As a result of the comprehensive rehabilitation program, the proportion of frail patients was significantly reduced by 9% (from 36.9% to 27.9%). The overall frailty index decreased by 0.4 points, driven by the significant changes of the single parameters cognition (MMSE), nutrition (MNA), and subjective mobility disability and mobility (TUG). On the other hand, it was small (N=136), short (observation for 3-4 weeks), observational type study without control group or randomization.

However, other authors have not confirmed the predictive value of this instrument and found that it only partly predicted unfavorable outcome after TAVI (nutrition and mobility) [41]. More studies with VHD patients and MGA should be done in order to receive higher level evidence, but its possibility of evaluating most of complex frailty syndrome components looks promising not only in screening but also in frailty dynamics after CR.

#### 3.1.3. Canadian Study of Health and Aging (CSHA) Scale or Clinical Frailty Scale or Rockwood Scale

CSHA scale is a multidomain approach that defines frailty as a proportion of accumulated health deficits [9, 26]. In the Canadian Study of Health and Aging, authors worked with 3 approaches: (1) developing a rules-based definition of frailty, (2) creating a method of counting a patient's clinical deficits, and (3) proposing the clinical frailty scale, a measure of frailty based on clinical judgment [42]. The seven-point scale is displayed in an easily visualized form including objective clinical judgment and subjective patient assessment [26]. It is a validated instrument for diagnosing physical frailty and easy to use [9, 42, 43]. Seiffert et al. used the Rockwood scale in the Bonn group (N=347/847 patients) undergoing TAVI and found frailty to be significantly related to 1-year mortality [26, 44]. Other authors also used this scale and reported significant results related to quality of life, poor physical and mental function, physical well-being [45], and poor outcomes even in pre-frail patients [46]. Shimura et al. compared it with other well-known frailty markers and concluded that CSHA predicted late mortality in an elderly TAVR patient cohort better than 5MWT, body mass index (BMI), and handgrip strength [47]. We did not find any study that would use CSHA scale to evaluate frailty dynamics after CR for patients with VHD. What is more, CSHA did not show superiority compare to other frailty tools (such as 5MWT or handgrip test) in other reviewed studies [24, 30]. On the other hand, CHSA scale is based upon patients level of disability, level of independence, that could be a link to other tools used in rehabilitation settings such as Barthel index (BI) or functional independence measure (FIM). Systematic review and meta-analysis performed by Ribeiro et al. [48] showed that frail patients after TAVI or surgical aortic valve replacement improved significantly according to BI or FIM results, compared before and after CR. What is more, recently published call of action regarding frailty in CR offered to use CSHA scale as possible frailty measure [9].

#### 3.1.4. 5-Meter Walking Test

One of the requirements for frailty assessment (especially for screening) is simplicity and ability to assess frailty in a very short period of time. One of the most evaluated frailty assessments is the 5-meter (15 feet ~ 4,5 meters) walking test which can be used as part of other scales (FFS) or as stand-alone evaluation [13, 20, 24, 27, 30, 47, 49–51]. This test results could be evaluated in different manner: (1) measuring time taken for 5-meter walk in seconds and (2) measuring gait speed in meters per second. Different authors offer different cutoff values to describe frail patients that vary from 5 to 7 seconds according to gender and body composition [6, 8]. Gait speed is an independent predictor of adverse outcomes after cardiac surgery, with each 0.1-m/s decrease conferring an 11% relative increase in mortality [52]. The slowest walkers (6-10 or more seconds) had 35% higher 30-day mortality than normal walkers (adjusted OR, 1.35; 95% CI, 1.01–1.80) [51]. Kano et al. described stepwise incremental increased risk of mortality after TAVI in patients with the slowest gait speed (<0.5 m/sec) or who were unable to walk compared to patients with a normal gait speed [50]. Studies that compared several frailty assessment tools also demonstrated 5MWT superiority among other more complex ways to evaluate frailty in VHD population [27, 53]. This test is validated in large trials, has good predictive value for patients undergoing cardiac surgery, and is superior to other instruments [12, 52]. Although it seems logical to use this 5MWT in cardiac rehabilitation, as it is recommended by AHA guidelines [54], none of the reviewed studies in CR setting chose this test. All of the mentioned studies regarding exercise training for patients with VHD evaluated six-minute walking test to show improvement in physical capacity, but this test failed to show predictive value in frailty screening before the procedure [55]. Future studies will be needed to evaluate 5MWT sensitivity in a setting of CR.

#### 3.1.5. Serum Albumin Levels

Pre-procedural albumin levels (as single measurement or as part of other indexes) have been demonstrated to have significant predictive value [47, 56–58]. Hypoalbuminemia (<3.5 g/dL) was associated with poor prognosis, highlighted by the increase in noncardiovascular mortality in elderly TAVI patients [58]. Grossman et al. showed that even higher pre-procedural albumin (< 4 g/dL) can significantly improve the ability of the Society of Thoracic Surgeons (STS) and EuroSCORE II scores to predict TAVR-related mortality [57]. In addition Bogdan et al. showed that low post-procedural (4 days after procedure) serum albumin remained a strong parameter correlated with all-cause mortality (HR=2.47; 95% CI: 1.284.78; p<0.01) [59]. Forcillo et al. analyzed whether MFFC predicted 30-day mortality after TAVI. The results demonstrated that although all MFFC components were associated with increased risk of adverse outcomes, only albumin (<3.5 g/dL) was predictive of 30-day all-cause mortality [28]. Eichler et al. focused on nutrition assessment but used another instrument, validated MNA scale [41]. Moreover, one of the components of the successful frailty risk score for the elderly undergoing aortic valve replacement (EFT) is low pre-procedural albumin level [24].

Albumin level seems to have higher prediction value and could be a good screening instrument for frailty. What is more, serum albumin levels can be linked to lower muscle mass in elderly [60]. Albumin as nutrition measure could be connected with low body mass index (BMI) and sarcopenia. Mok et al. showed that higher BMI in TAVI patients was associated with higher skeletal muscle mass [61]. However, 46% of these cases were sarcopenic obesity that was significantly higher than the 4% to 12% in the general population [61]. These results could give an impression that muscle mass is more important than whole body mass. Shimura et al. showed significant relation between lower BMI and higher level of frailty [47]. The results from Koifman et al. confirmed that BMI <20 kg/m2 should be considered as a frailty marker during the screening process of severe aortic stenosis TAVI patients as it is associated with higher mortality [62]. Albumin levels should be taken in to account during comprehensive CR program, which is the role of dietician counseling, helping to reach healthy body mass not only through healthy diet but also through increasing muscle mass.

#### 3.1.6. Katz Index of Activities of Daily Living

The Katz index is used for functional assessment of dependency in elderly individuals in the six functions: feeding, bathing, dressing, transferring, toileting, and urinary continence [95]. It provides objective data on a patient's independency in daily activities [26]. Frailty usually overlaps with disability; hence some authors use the same instruments for disability measurement while others use it for frailty assessment [32, 69]. Earlier studies incorporated Katz index in the frailty assessment and demonstrated that it is an independent risk factor for in-hospital mortality and institutional discharge in patients after cardiac surgery [15, 26, 96]. Therefore, a more pragmatic approach in defining frailty, commonly called the social domain, often includes an ADL assessment [26]. Puls et al. identified a Katz index <6 as a powerful independent predictor of immediate-, short-, and long-term all-cause mortality in TAVI patients [26, 77]. Most of the review studies included Katz index as part of frailty assessment and demonstrated it to produce good prediction values [17, 49, 68, 69, 72]. Recently published American College of Cardiology/American Heart Association (ACC/AHA) and European Society of Cardiology (ESC) guidelines recommend the Katz index as one of the frailty assessment instruments for risk prediction before cardiac surgery [97, 98]. On the other hand, other authors did not detect Katz index as good predictor for long- and short-term outcomes; in those studies it was inferior to mobility and nutrition measures [30, 41, 64]. Although usage of Katz index looks promising, we failed to find any study in CR with VHD patients that would test individuals with KI before and after CR program. Eichler et al. used IADL and basic DAL [40]; other authors successfully used BI or FIM to evaluate frailty, disability, or patient independence [85, 99].

### 3.2. What Exercise Interventions Would Be the Most Beneficial for Frail Patients?

In order to answer the second research question, we analyzed 12 studies published in English since 1980 on topic of VHD and exercise training with elderly (Table 2). All analyzed studies demonstrated benefit of exercise training in patients after surgical/interventional VHD treatment. Although the evaluated exercise-based CR programs were differently structured, the patient population included and the primary endpoints examined were similar and focused on the results of 6-minute walking test and cardiopulmonary stress test (peak VO_2_). 6 studies were conducted in inpatient facilities and 6 studies analyzed the results of outpatient programs only. In 8 studies the program started early (< 1 month) after surgery or intervention. Other studies included patients after ≥1 month of surgery or intervention. In all reviewed publications specified documented adverse events were not related to exercise [91, 93, 100].

Although recently published studies concentrate on disabled, comorbid high-risk elderly patients, all authors demonstrated that exercise training is safe and effective in improving functional and physical capacity [40, 84, 85, 87, 89, 93], quality of life [40, 84], and independence in daily life activities [48, 84, 85, 87, 93]. Exercise training type, method, length, and intensity differed among the studies and did not allow clear recommendations concerning how exactly this patient group should be treated.

Only two randomized controlled trials (RCT) were performed with patients aged 60 years or older [91, 92]. Low sample size pilot study on exercise training after TAVI, with combined endurance and resistance training, showed significant improvements in exercise capacity, muscular strength in different muscles groups, and quality of life compared with usual care [91]. In this study, 13 TAVI patients participated in an eight-week outpatient CR, starting 2-3 months after intervention. Resistance training was added in the second week of the program and performed in 2 of the 3 weekly workouts. It included 5 different exercises starting with 1 set with 10 repetitions at 30% 1 repetition maximum (1-RM) and was gradually increased to 3 sets with 15 repetitions at 50% to 60% 1-RM. Sbilitiz et al. showed that CR group compared with control had a beneficial effect on VO2 peak at 4 months (24.8 mL/kg/min versus 22.5 mL/kg/min, p=0.045) but mental health and other measures of exercise capacity and self-reported outcomes was not affected [92]. Exercise training in this study was initiated 1 month after surgery, comprising 3 weekly exercise sessions for 12 weeks. The program consisted of graduated cardiovascular training and strength exercises.

None of these studies assessed level of patients frailty before and after exercise training program; on the other hand, it did show that structured training program could improve older patient physical capacity [91, 92]. What is more, Sbilitiz et al. did not find improvement in patients' mental health which could be seen as a one component of complex frailty syndrome. It is interesting that latter study had special structured psychosocial education that cover relevant topics: changed body-image and self-image, recovery from major surgery, and dependency on relatives and medical issues, but did not receive positive results [92].

Only one study, conducted by Eichler et al., evaluated the effect of a multicomponent inpatient CR on frailty in patients after TAVI [40]. It is important to mention that the CR included patient education, diet counseling, psychological support, and risk factor management as well as individualized training components. Exercise training performed was mainly aerobic such as cycle-ergometer training and Nordic walking. Patients with a higher exercise capacity (> 1.0 W/kg) conducted additional strength training at 30–50% 1-RM. For frailty assessment, frailty index by Schoenberg et al. was used. As a result of the rehabilitation the proportion of frail patients was significantly reduced by 9% (from 36.9% to 27.9%). The overall frailty index decreased by 0.4 points, driven by the significant changes of the single parameters cognition (MMSE), nutrition (MNA), and subjective mobility disability and mobility (TUG). Although frailty index could be reversed during cardiac rehabilitation, it did not show a prognostic impact itself [40]. On the other hand, it was observational type study, did not have control group, and was not designed as randomized control trial.

Russo et al. showed that early cardiac rehabilitation enhances independence evaluated with Barthel index, mobility, and functional capacity for patients after surgical or interventional aortic stenosis treatment. The exercise training consisted of aerobic training and calisthenics [85]. Systematic review and meta-analysis performed by Ribeiro et al. [48] showed that patients after TAVI or surgical aortic valve replacement achieve similar benefits from cardiac rehabilitation. Frailty components analyzed in this review were independently measured by Barthel index or functional independence measure (FIM), which improved significantly in both groups. The cardiac rehabilitation programs included exercise endurance training (cycle or treadmill), resistance training, and/or inspiratory muscle training.

Summarizing of existing evidence on exercise training for patients with VHD would show that exercise program should be individualized and intensity should be measured by RPE with beginning of 40-60% of maximal VO2. All analyzed training protocols included aerobic training component implemented through various activities (Nordic walking, bicycle ergometer, gymnastics, etc.) and in 9 of them additional strength training was performed starting form the second or third week of exercise training, measuring intensity with 1 repetition maximum method. Inpatient CR program length varied from 2 to 4 weeks, while the duration of the outpatient programs was 12-24 weeks.

### 3.3. Would the Change of Frailty Status Improve Outcomes on Levels of Disability, Functional Capacity, and Quality of Life after Surgical/Interventional VHD Treatment beyond the Effects of the Surgery/Intervention Itself?

According to recent studies, in 23-32,8% of cases functional capacity of TAVI patients did not improve or even deteriorate in a period of 6-12 months after intervention [101–104]. If we put aside certain procedure technique, surgeon experience, risk-benefit ratio evaluation, and careful patient selection, individualized care or CR in inpatient/outpatient setting could outweigh unwelcome complications. Older VHD patients constitute a sensitive group that requires more specific comprehensive cardiac rehabilitation programs and they return to casual life could be more difficult [4, 105].

After analyzing the reviewed studies, we could not directly answer to the third research question. Two studies that were designed as RCT did not evaluated frailty or its components [91, 92], and study that was designed to evaluate exercise training impact on frailty did not have control group [40]. On the other hand, all reviewed studies show positive impact of CR and did not register any adverse event or clinical deterioration linked to exercise training program.

Participation in CR improves outcomes of patients with cardiovascular disease [106] and may be specifically beneficial for patients with frailty syndrome; however, cardiac rehabilitation facility remains unused.

## 4. Discussion

The results of this review highlight the importance of frailty syndrome in patients with VHD. Screening for frailty as a high-risk factor is well implemented in Heart team decision making for high-risk patients with aortic stenosis. However, there is gap of evidence considering frailty in CR settings. This literature review was designed to seek for answers of several research questions: Are assessment tools used for frailty screening sensitive enough to show improvement in frailty status after exercise-based CR? What exercise interventions would be the most beneficial for frail patients? Would the change of frailty status improve outcomes on levels of disability, functional capacity, and quality of life after surgical/interventional VHD treatment beyond the effects of the surgery/intervention itself?

First of all, our analysis revealed several different methods used for frailty assessment and evaluation. The analysis of these frailty tools was difficult because (1) a huge variety of instruments were used, (2) the same instruments were chosen to evaluate disability or cognitive functions in some studies and frailty in others, (3) the same instruments were named differently in separate studies, (4) authors were using validated scales but chose to analyze components separately, (5) authors created and offered new risk scores for frailty assessment that has not been implemented in other studies, and (6) some studies described subjective frailty evaluation manner “eye-ball test” [78, 79], while others used objective, quantitative or semiquantitative tests to evaluate all possible deficits.

It is clear that the prevalence of frailty plays an important role for patients with VHD: recently updated AHA VHD and ESC guidelines emphasize the frailty issue as an important factor for Heart team decision making in patients with aortic stenosis [97, 107]. In 2014, the AHA guidelines recommended the use of Katz Index of independence of daily living and the 5-meter gait speed test, and this recommendation was left in 2017 [54, 107]. Frailty as worse outcome predictor is already mentioned in ESC guidelines in 2012, but still without clarification of how exactly frailty should be measured and evaluated [108]. ESC 2016 guidelines indicate that frailty assessment should not be subjective and criticize the “eyeball test” [97]. According to cited studies frailty could be measured with complex evaluation of the patient that includes Katz index of independence of daily living, cognitive function evaluation, nutrition assessment, and patient functional capacity (TUG) [97].

Existing literature confirms frailty predictive value for higher complications rate, longer length of hospital stay, and mortality and morbidity after cardiac surgery or interventional treatment [9, 97]. On the other hand, most of the studies describe frailty assessment before procedure. Frailty status is dynamic and can transit over time. In most cases people become more frail while aging, but in up to 20% of the population frailty criteria also can disappear over time [109, 110]. Study that analyzed community living elderly persons showed that, of the 754 participants, 434 (57.6%) had at least 1 transition between any 2 of the 3 frailty states during 54 months. Transitions to states of greater frailty were more common (rates up to 43.3%) than transitions to states of lesser frailty (rates up to 23.0%) [109, 110]. A small study that was performed by Forman et al. showed significant worsening of frailty level (measured by CSHA, 5MWT, and MMMSE) and functional capacity in 10 weeks for patients that are waiting for TAVI [111]. This dynamics shows that even short-term (3-4 weeks) CR could be effective for these patients.

Vigorito et al. recently published a call for action on frailty in CR and offer mainly two instruments for frailty assessment in this specific field: Edmonton frailty scale (EFS) and clinical frailty scale from the CSHA study. The EFS could be used as a comprehensive instrument that has been validated in elderly patients after acute coronary syndromes [9]. It also includes two clinical performance items interrogating cognition (clock test) and functional performance (TUG) [9]. However, some EFS questions are developed for screening more than for effect evaluation (e.g., hospitalizations per year, functional independence, social support, and medication usage) and would not be sensitive to show results of comprehensive CR. Also we did not find any study with VHD patients where frailty would be measured with EFS. Clinical frailty scale from the CSHA is popular and looks effective for screening in this patient population [20, 35, 44]. It is easy-to-use standardized tool based on subjective evaluation of frailty; however, it lacks psychosocial factors [9, 26]. Moreover, studies that compare different frailty tools (including CSHA frailty scale) did not show its superiority to other instruments for frailty screening and outcomes prediction [24, 27, 30].

Our analysis revealed the six most used tools for frailty in VHD population: 3 scales (fried frailty scale and its modifications, multidimensional geriatric assessment, and clinical frailty scale from the CSHA study) and 3 single measures (5-meter walking test, Katz index, and serum albumin levels). Together with existing guidelines and expert opinions we believe that simple and fast-performed single test would not show comprehensive CR effect on a frail patient. An instrument of frailty assessment in CR should have the various components included in different risk assessment scores (mobility, independence in daily living, cognitive functions, nutrition, muscle mass and strength, and anxiety and depression evaluation). We did not find enough studies performed in CR with frail patients to find out which of all the mentioned tools is the best and most sensitive for frailty syndrome and its dynamics after CR program. Future studies are needed to build new evidence in this field of research.

Second research question aim was to find an ideal exercise training-based CR program for frail patient with VHD. It is known that exercise-based comprehensive CR is based on strong evidence and is a well-recognized treatment for patients with different cardiovascular diseases [112, 113]. Physical rehabilitation for frail older people can positively affect functional and physical capacity and this effect may be related to the level of frailty [23]. Physical activity recommendations and supervised exercise programs can be useful for frailty prevention, to improve physical and functional capacity (gait speed, TUG, and SPPB), survival, and quality of life as well as to decrease prevalence of anxiety and depression and risk of falls in community-dwelling frail older people [114–117]. According to recent published systematic review, interventions for pre-frail and frail older adults should include multicomponent exercises, including in particular resistance training, as well as aerobic, balance, and flexibility tasks [117]. A study that included older patients soon after CABG demonstrated that additional resistance training and special balance training improved functional capacity (6-MWD, TUG time) significantly more than aerobic training alone [118]. Exercise training-based cardiac rehabilitation could be a treatment option for frail VHD patients. EAPC Cardiac Rehabilitation Section raised awareness and importance of frailty topic in the field of CR [9].

However, literature data in exercise training and CR in VHD patients is scare [100]. According to a published Cochrane meta-analysis, exercise training is beneficial and provides short-term improvement; however, it was assessed by only two studies included in this meta-analysis, and both of them were performed in a young-middle aged sample [100]. Our literature review showed that different exercise training programs with VHD elderly patients after surgery/intervention were safe and effective in improving functional and physical capacity [40, 84, 85, 87, 89, 93], quality of life [40, 84], and independence in daily life activities [48, 84, 85, 87, 93].  Studies with frail patients demonstrated that exercise-based programs increased gait speed and improved balance and performance in activities of daily life or SPPB [9]. A systematic review on exercise-based CR for patients after aortic stenosis surgical/interventional treatment demonstrated that a short-term (2-6 weeks) program could improve Barthel index, FIM, 6MWT, and anxiety and depression [48]. Eichler et al. demonstrated that participating in comprehensive CR results in frailty index decrease by 0.4 points, driven by the significant changes of single parameters such as cognition (MMSE), nutrition (MNA), and subjective mobility disability and mobility (TUG). In addition, several studies showed improvement in frailty and physical and functional capacity in frail patients, although baseline evaluation before surgery/intervention demonstrated lower results in the frail patient group [18].

All reviewed studies had an aerobic component of exercise training that was fulfilled in different ways, and 9 of 12 reviewed studies had an additional strength-training component. All described exercise programs were individualized according to the patient capability and described careful exercise intensity dosage according to rate of perceived exertion. We believe that ideal CR for VHD patients should be supervised by a cardiologist, and its content should include (1) exercise training (endurance and strength training to improve muscle mass, strength, balance, and coordination and to avoid falls), (2) nutrition counseling (to improve healthy body mass), (3) occupational therapy (to improve independency and cognitive function), (4) psychological counseling to ensure psychosocial health, and (5) social worker counseling (to improve independency). To improve outcomes of these patients CR services need to be optimally utilized and the protocols modified to cater for frail patients and to monitor their progress over the course of the treatment [106].

## 5. Limitations

Our literature review has several limitations and weaknesses. First of all, it was not designed as a meta-analysis or systematic review, so results of our study have lower evidence level. We searched only one database and used informal and subjective methods for studies inclusion and quality check. A quality assessment of the included studies would have strengthened our study.

What is more, we failed to find concrete and practical answers for research questions that were raised in the beginning of the study. We could only provide existing situation and try to find possible goals for future studies.

## 6. Conclusions

Frailty assessment in CR settings should be based on functional, objective tests and should have similar components as tools for risk assessment (mobility, muscle mass and strength, independence in daily living, cognitive functions, nutrition, and anxiety and depression evaluation). Participating in comprehensive exercise-based CR could improve short- and long-term outcomes in frail VHD patient. Comprehensive CR not only should include exercise training, psychological interventions, and improvement in nutrition but also could prevent, restore, and reduce the severity of frailty as well as improving outcomes for frail VHD patients.

## Figures and Tables

**Table 1:** Characteristics of reviewed studies on frailty and VHD.

**Publication** **(year)**	**Sample size (N) Patient population (Age, Female N (**%**))**	**Intervention Frail sample N (**%**)**	**Tool for frailty assessment**	**Primary end point**	**Main results/Conclusions**
**Abramowitz (2016) **[29]	N=805; Age 82.0±8.8, F 321 (39,9%)	TAVI 279 (34.7%)	(1) MFFC (2) BMI	(1) To assess the influence of BMI on the short- and midterm clinical outcome following TAVI.	(1) Obese patients had lower prevalence of frailty. (2) All-cause mortality up to 30 days was 2.9% (10/340) vs 4.5% (12/268) vs 0.5% (1/186) in patients with normal weight, overweight, and obesity, respectively (p<0.048). (3) In a multivariable model, overweight and obese patients had similar overall mortality compared to patients with normal weight.

**Ad ** **(2016)** [33]	N=167; Age 74.1±6.6, F 41 (25%)	Cardiac surgery 39 (23%)	(1) FFS	(1) Impact of frailty on outcomes of patients undergoing cardiac surgery	(1) Frail patients had longer median ICU stays (54 vs. 28h, p=0.003), longer median LOS (8 vs. 5 days, p<0.001), greater likelihood of STS-defined complications (54% vs. 32%, p=0.011), and discharge to an intermediate-care facility (45% vs. 12%, p<0.001) (2) not different from non-frail patients on major outcome, operative mortality, or readmissions.

**Afilalo ** **(2010)** [6]	N=131; Age 75.8± 4.4; F 44 (34%)	Open cardiac surgery 60 (46%)	(1) 5MWT	(1) Inhospital post-operative mortality or major morbidity, defined by the STS.	(1) Slow gait speed was an independent predictor of the composite end point after adjusting for the STS risk score (OR: 3.05; 95% CI: 1.23 to 7.54).

**Afilalo** **(2012)** [12]	N=152; Age 75,9±4,4, F 52 (34%)	Cardiac surgery 20%- 46%	(1) FFS (2) 7-item FFS (3) 4-item MSSA (4) 5MWT	(1) The STS composite end point of in-hospital postoperative mortality or major morbidity	(1) The most predictive scale in each domain was 5MWT (2) ≥6 s as a measure of frailty

**Afilalo ** **(2016)** [52]	N=15171; Age 71, F 4622 (30.5%).	Cardiac surgery 4588 (30,24%)	(1) 5MWT	(1) Operative mortality or within 30 d	(1) Compared with patients in the fastest gait speed tertile, operative mortality was increased for those in the middle tertile (0.83-1.00m/s; OR, 1.77; 95% CI, 1.34-2.34) and slowest tertile. (2) After adjusting gait speed remained independently predictive of operative mortality (OR, 1.11 per 0.1-m/s decrease in gait speed; 95% CI, 1.07-1.16). (3) Gait speed was also predictive of the composite outcome of mortality or major morbidity (OR, 1.03 per 0.1-m/s decrease in gait speed; 95% CI, 1.00-1.05).

**Afilalo** **(2017)** [24]	N=1020; Age 82 (77–86), F 421 (41%)	SAVR, TAVI 26-68%	(1) FFS (2) MFFC (3) CHSA (4) SPPB (5) Bern scale (6) Columbia scale (7) EFT	(1)Death from any cause at 12 mo (2) death from any cause at 30 d or worsening and institutionalization (3) new accrued disabilities at 12 mo	(1) EFT was the strongest predictor of death at 1 y (adjusted [OR]: 3.72; 95% [CI]: 2.54 to 5.45). (2) EFT was the strongest predictor of worsening disability at 1 y (adjusted OR: 2.13; 95% CI: 1.57 to 2.87) and death at 30 d (adjusted OR: 3.29; 95% CI: 1.73 to 6.26).

**Alfredsson ** **(2016)** [51]	N=8039; Age 84 (79–88) F 4128 (51.4%)	TAVI 6100 (75,88%)	(1) 5MWT	(1) all-cause mortality at 30 d	(1) 30-d all-cause mortality rates were 8.4%, 6.6%, and 5.4% for the slowest, slow, and normal walkers, respectively (P<0.001). (2) Each 0.2-m/s decrease corresponded to an 11% increase in 30-d mortality (adjusted OR, 1.11; 95% CI, 1.01–1.22). (3) The slowest walkers had 35% higher 30-d mortality than normal walkers (adjusted OR, 1.35; 95% CI, 1.01–1.80), significantly longer LOS, and a lower probability of being discharged to home.

**Arnold ** **(2017)** [32]	N=2830; Age 83.3, F 1284 (45.4%)	TAVI 1692 (59.8%)	(1) FFS	(1) death within the first 6 mo after TAVI; (2) very poor QoL at 6 mo; (3) moderate worsening in QoL from baseline to 6 mo	(1) For all models except the 1-year clinical model, frailty was associated with an increase in the odds of a poor outcome of 30% to 40% when added to the existing models; (2) Adding frailty as a syndrome increased the c-indexes by 0.000 to 0.004, with the most important individual components being disability and unintentional weight loss

**Assmann** **(2016)** [19]	N=89; Age 80.4±6,3, F 51 (57%)	TAVI NA	(1) MGA/MGBE	(1) Postprocedural period, mortality in 30 d follow-up	(1) Variables from frailty assessment protectively associated with delirium were MMSE, IADL and gait speed (2) TUG was predictively associated with delirium (3) MMSE was independently associated with delirium. (4) Variables predictively associated with mortality were the summary score Frailty Index (HR 1.66, 95% CI 1.06 to 2.60; p=0.03) (5) Variables from frailty assessment are associated with delirium and mortality

**Bagienski** **(2017)** [20]	N=141; Age 82.0, F 89 (63,1%)	TAVI 2,8-90,1%	(1) 5MWT, (2) Elderly mobility scale (3) CSHA, (4 ) Katz index, (5) Grip strength, (6) ISAR	(1) All-cause mortality at 30 d and 12 mo	(1) 30-d and 12-mo all-cause mortality rates were higher in the delirium group (p <0.001). (2) Significantly more patients with delirium were considered as frail before TAVI.

**Bogdan ** **(2016)** [59]	N=150; Age 81±6 years F 90 (60%)	TAVI 79 (53%)	(1) Low albumin	(1) Correlation between baseline serum albumin and all-cause mortality in TAVI patients. (2) low post-procedural albumin following TAVI.	(1) Mortality was higher in the low albumin group compared with the normal albumin group (35% vs. 19%, p=0.01). (2) Multivariate analysis indicated that low preprocedural albumin (≤40 g/L) was independently associated with a more than twofold increase in 2.1 year all-cause mortality (p=0.01, HR=2.28; 95% CI: 1.174.44). (3) Low postprocedural serum albumin remained a strong parameter correlated with all-cause mortality (HR=2.47; 95% CI: 1.284.78; p<0.01).

**Capodanno (2014)** [63]	N=1878 NA F 1087 (57,9%)	TAVI 459 (24,4%)	(1) Geriatric status scale	(1) 30-d mortality	(1) Frailty OR 2.09, CI (1.30-3.37), p =0.003 (2) OBSERVANT risk model did not include frailty as a risk factor

**Chauhan ** **(2016)** [38]	N=342; Age 81.85, F 179 (52,3%)	TAVI 104 (30%)	(1) MFFC	(1) all-cause mortality (2) total postoperative LOS, discharge disposition and incidence of stroke.	(1) Patients with frailty score of 3/4 or 4/4 had increased all-cause mortality (P = 0.015 and P <0.001) and were more likely to be discharged to an ICU facility (P =0.083 and P = 0.001). (2) 4/4 frail patients had increased post-operative LOS (P = 0.014) (3) Individual components of the frailty score were also independent predictors of all-cause mortality. (4) The HR of mortality increased with each increase in frailty score

**Cockburn** **(2015)** [64]	N=312; Age 81.2±6.8, F 146 (46,8%)	TAVI NA	(1) General mobility EuroSCORE II (2) Brighton mobility index (3) NYHA (4) Karnofsky performance scale (5) Katz Index (6) CSHA	(1) Assess whether different frailty indices predict outcomes both in the shorter and longer terms	(1) Both univariate and multivariate analyses confirmed poor mobility (EuroSCORE II), as the best predictor of adverse outcome over both the short-term (OR 4.03, 95% CI (1.36–11.96), P50.012 (30 days)) and longer term (OR 2.15, 95% CI (1.33–3.48), P50.002, (2.261.5 years.) (2) Mobility impairment, of either neurological or musculoskeletal etiology, is an appropriate screening measure when considering patients for TAVI.

**Codner** **(2015)** [49]	N=360; Age 82. ± 6.9, F 203 (56.4%)	TAVI NA	(1) 5MWT (2) Katz Index, (3) albumin levels, (4) oxygen therapy, (5) cognitive status, (6) gen. appearance	All-cause mortality during follow-up.	In multivariate analysis frailty (HR 1.89, 95% CI 1.11 to 3.2, p =0.02) was independent predictor for all-cause mortality.

**Dahya ** **(2016)** [65]	N=104, Age 81 ± 10, F 50 (48%)	TAVI NA	CT scan – SMI MFFC	(1) Relationship between SMI and LOS	(1) A multivariate model showed SMI as independent predictors of LOS. (2) For every 14-cm2/m2 increase in SMI, there was a 1-d reduction in LOS. (3) None of the standard measures of frailty predicted LOS.

**de Thézy** **(2017)** [66]	N=49; Age 84.8, F 27 (55.1%)	TAVI 41 (83.67%)	(1) G8 scale (2) CGA	(1) sensitivity and specificity of G8	(1) G8 had a sensibility of 100% (IC 95% [0.91]), a specificity of 72.7% (IC 95% [0.430.9]), a positive predictive value of 92.6% and a negative prospective value of 100% (IC: 95%). (2) G8 scale could be performed by cardiologists in older patients with AS for identifying patients with a geriatric risk profile in consultation before surgery.

**Debonnaire (2015)** [37]	N=511 Age 82 F 317 (62%)	TAVI 98 (21%)	(1) FFS (2) Eye ball test	(1) 1y mortality	(1) Frailty was not associated with the study end point

**Eichler** **(2017)** [41]	N=344; Age 80.9 ± 5.0; F 191 (55,5%)	TAVI 152 (45.8%)	(1) MGA/MGBE	(1) All-cause mortality at 12 mo after TAVI.	(1) MGA/MGBE had no predictive power; its individual components, particularly nutrition (OR 0.83 per 1 pt., CI 0.72–0.95; p=0.006) and mobility (OR 5.12, CI 1.64–16.01; p=0.005) had a prognostic impact.

**Esses** **(2018)** [43]	N= 3088; Age 69.24 ± 13.10, F 1163 (37.7)	SAVR 25,74- 56,19%	(1) modified CSHA (2) RAI (3) 6-mo mortality index by Porock (3) Ganapathi index	(1) 30-d mortality and major postoperative morbidity.	(1) Frailty was a better predictor of mortality than morbidity, and it was not markedly different among any of the 3 indices. (2) Frailty was associated with an increased risk of 30 d mortality and longer LOS.

**Forcillo** **(2017)** [28]	N=361; Age 82; F 167 (43%)	TAVI NA	MFFC	(1) 30-d mortality and to compare the discrimination of 30-d mortality, and to compare its discriminative ability with STS PROM.	(1) For high- and extreme-risk patients undergoing TAVR, serum albumin, Katz Index, and 5MWT were associated with increased risk of adverse outcomes. (2) Only albumin was predictive of 30-day all-cause mortality.

**Garg ** **(2017)** [67]	N=152; Age 83.3±6.5, F 64 (42%)	TAVI 76 (50%)	(1) CT scan - PMA	(1) early poor outcome (30 d mortality, stroke, dialysis, and prolonged ventilation	(1) Indexed PMA ([OR] 3.19, [CI] 1.30 to 7.83; p =0.012) and age (OR 1.92, CI 1.87 to 1.98; p = 0.012) predicted early poor outcome. (2) High-resource utilization was observed more frequently in patients with PMA less than the median (73% vs 51%, OR 2.65, CI 1.32 to 5.36; p = 0.006).

**Goldfarb ** **(2017)** [31]	N=235 Age 73.0; F 68 (29%)	Cardiac surgery N=91, (38,72%)	FFS or SPPB	(1) The impact of preoperative frailty status on postoperative hospitalization costs	(1) The median cost was $32,742 in frail patients compared with $23,370 in non-frail patients (P < 0.001). (2) Total costs were independently associated with frailty and valve surgery (P < 0.001). (3) This effect persists after adjusting for age, sex, surgery type, and surgical risk score.

**Green** **(2013)** [55]	N=484; Age 84,7, F 224 (46%)	TAVI 351 (72,52%)	(1) 6MWT	(1) association between baseline 6MWT and functional improvement (2) association between baseline 6MWT and mortality.	(1) There were no differences in 30-d outcomes among 6MWTD groups. (2) At 2 y, the rate of death from any cause was 42.5% in those unable to walk, 31.2% in slow walkers, and 28.8% in fast walkers (p = 0.02), driven primarily by differences in noncardiac death. (3) Patients with poor baseline functional status exhibit the greatest improvement in 6MWTD.

**Green** **(2015)** [39]	N=244; Age 86,25, F 1118 (48,3%)	TAVI 110 (45%)	(1) MFFC	(1) Time to death from any cause over 1 y of follow up and poor outcome at 1y	(1) At 30 d, there were no differences in rates of MACCE according to baseline frailty status. (2) At 1 y all-cause mortality rate was 32.7% in the frail group and 15.9% in the non-frail group (log-rank p=0.004). (3) Frailty remained independently associated with an increased odds of poor outcome after TAVI at both 6 mo (OR 2.21, 95% CI 1.09–4.46, p = 0.03) and 1 y (OR 2.40, 95% CI 1.14–5.05, p = 0.02).

**Grossman ** **(2017)** [57]	N=426; Age 83.8, F 242 (56,8%)	TAVI 192 (45,07%)	(1) Low albumin	(2) 1-y all-cause mortality.	(1) Participants with low albumin levels had higher mortality (HR) = 3.03, 95% (CI) = 1.66–5.26, P < .001). (2) Participants with low serum albumin and a high STS (HR = 4.55, 95% CI = 2.21–9.38, P < .001) or EuroSCORE-2 (HR = 2.72, 95% CI = 1.48–5.06, P = .001) (3) Serum albumin, as a marker of frailty, can significantly improve the ability of STS and EuroSCORE-2 scores to predict TAVR-related mortality.

**Herman** **(2013)** [68]	N=4270; Age 67, Female 25%	Cardiac surgery 171 (4%)	(1) Katz index (2) independence in ambulation (3) dementia	(1) Composite end point defined as MACCE	(1) Frailty was significant (OR 1.7; 95% CI 1.2-2.5) predictor. (2) The concordance statistic for the MACCE model in a mixed population was 0.764 (95% CL; 0.75-0.79) and had excellent calibration.

**Hermiller** **(2016)** [69]	N=3687; Age 83.3 ± 7.8 F 1707 (46.3%)	TAVI NA	(1) 5MWT (2) grip strength (3) BMI (4) anemia (5) hypoalbuminemia (6) weight loss; (7) Fall	All-cause mortality rate 30 d and 1 y	(1) Albumin levels <3.3 g/dl predicted death at 30 d. (2) Albumin levels <3.3 g/dl, falls in the past 6 months, predicted death at 1 y. (3) This score (Albumin, assisted living, home oxygen, age>85y) showed a 3-fold difference in mortality rates for the low-risk and high-risk subsets at 30 d (3.6% and 10.9%, respectively) and 1 y (albumin, comorbidities, home oxygen, STS>7%) (12.3% and 36.6%, respectively).

**Huded** **(2016)** [36]	N=191 Age 82.4 ± 9.2; F 93 (49%)	TAVI N=64, (33,5%)	FFS	(1) 30-d mortality, AE, hospital readmission (2) hospital LOS, (3) Discharge to a rehabilitation facility,	(1) There was no difference in 30-d mortality, major complications, mean hospital LOS, 30-day hospital re-admission, or overall survival between groups. (2) Frailty was independently associated with discharge to a rehabilitation facility (p=0.004).

**Kamga** **(2013)** [70]	N=30; Age 86 ± 3, F 14 (47%)	TAVI NA	(1) The ISAR (2) The SHERPA score	(1) Outcomes 30 d and 1 y	(1) The ISAR score was similar but the SHERPA score was significantly higher in non-survivors (7.8 ±1.6 vs. 4.9 ±2.4; P = 0.001). (2) SHERPA score (>7) and BMI were independent predictors of 1y mortality (P = 0.004).

**Kano ** **(2017)** [50]	N=1256; Age NA F 895 (71,25%)	TAVI 693 (55,17%)	(1) 5MWT	(1) 30 d and 12 mo mortality	(1) The slowest walkers and those unable to walk demonstrated independent associations with increased midterm mortality after adjustment (HR, 1.83, 4.28; 95% CI, 1.03–3.26, 2.22–8.72; P=0.039, <0.001, respectively). (2) Gait speed <0.385 m/sec associated with worse prognosis (HR 2.40; 95% CI, 1.75–5.88; P=0.001). (3) Increased midterm mortality rate in patients with a gait speed of 0.385~0.5 m/sec, or unable to walk as compared with patients with a normal gait speed.

**Kleczynski** **(2017)** [30]	N=101; Age 81.0 F 60.4%	TAVI 6,9-52,5%	(1) Katz index (2) EMS (3) Katz Index (4) CSH (5) 5MWT (6) Hand grip strength (7) ISAR	12-mo mortality	(1) Associations between frailty indices and 12-mo all-cause mortality were significant, adjusted for logistic EuroSCORE: (1) for 5MWT, 72.38 (15.95-328.44); (2) for EMS, 23.39 (6.89-79.34); (3) for CSHA scale, 53.97 (14.67-198.53); (4) for Katz index, 21.69 (6.89-68.25); (5) for hand grip strength, 51.54 (12.98-204.74); (6) for ISAR scale, 15.94 (2.10-120.74). (2) 5MWT, EMS, or hand grip test may be advocated.

**Kobe ** **(2016)** [71]	N=130; Age 83.3 F 65 (50%)	TAVI 71 (55%)	(1) FORECAST	(1) 12-mo mortality	(1) ROC showed that the FORECAST is a valid tool to predict in-hospital mortality (area 0.73). (2) By combining the FORECAST and the STS score, this effect was even higher (area 0.77; P = 0.021). (3) Stratifying the patients according to the FORECAST score showed best survival in the lowest frailty group.

**Koifman ** **(2016)** [62]	N=491; Age 83,8 F 245 (50%).	TAVI 43 (8,75%)	BMI	(1) all-cause mortality at 1 y of follow-up	(1) All-cause mortality at 1 y was higher in the low-BMI group (log-rank p=0.003) with no significant difference among normal and above-normal BMI patients. (2) In a multivariate model, BMI <20 kg/m2 was an independent predictor of mortality (HR=2.45, p=0.01)

**Kotajarvi ** **(2017)** [45]	N=103; Age 80.6 years, F 42 (40,1%)	SAVR 54 (52,4%)	(1) CSHA	(1) to determine the extent to which surgery affected measures of physical and mental health and QoL, (2) examine how changes in these patient-centered outcomes compared between non-frail and frail study participants.	(1) Frail participants had lower baseline independence and QoL measures; (2) At follow-up, frail participants showed significant improvement in physical function, with physical health scores improving by 50% and 14%. Non-frail subjects did not significantly improve in these measures. (3) Mental health scores also improved to a greater extent in frail participants (3.6 vs < 1 point). (4) Frail participants improved to a greater extent in physical well-being (21.6 vs 7.1 points) and quality of life measures (25.1 vs 8.7 points)

**Laure Bureau (2017)** [72]	N=116; Age 86.2 ± 4.2 F 116 (49.1%)	TAVI 10-58%	MPI score the sum of (1) Katz Index (2) MNA-SF (3) SPMSQ (4) CIRS	All cause of mortality at 1 mo	Mortality rate was significantly different between MPI groups at 6 and 12 mo (p=0.040 and p=0.022). Kaplan Meier survival estimates at 1 y stratified by MPI groups were significantly different (HR=2.83, 95% (CI) 1.38–5.82, p=0.004). Among variables retained to perform logistic regression analysis, Katz index appeared the most relevant (p < 0.001).

**Lee** **(2010)** [15]	N=3826; Age 68,5; F 998 (26%)	Cardiac surgery 157 (4.1%)	(1) Katz index (2) independence in ambulation (3) dementia	(1) In-hospital mortality, midterm all-cause mortality and discharge to an institution	(1) Frailty was an independent predictor of in-hospital mortality (OR 1.8, 95% CI 1.1 to 3.0), as well as institutional discharge (OR 6.3, 95% CI 4.2 to 9.4). (2) Frailty was an independent predictor of reduced midterm survival (HR 1.5, 95% CI 1.1 to 2.2).

**Mamane** **(2016)** [73]	N=208; Age 80.7± 6.8 F 114 (55%)	TAVI NA	(1) CT scan – PMA	All-cause mortality	(1) PMA was lower in non-survivors compared with survivors among women (12.9 vs 14.5 cm2; P = 0.047) but not men (21.7 vs 22.4 cm2; P = 0.50). (2) The association between PMA and all-cause mortality in women persisted after adjustment (HR, 0.88 per cm2; 95% CIerval, 0.78-0.99). (3) PMA is a marker of frailty associated with midterm survival in women

**Martin** **(2017)** [74]	N=6339; Age, 81.3; F 2927 (46.2%)	TAVI NA	(1) Katz Index (2) CSHA (3) poor mobility	(1) Internally validate a multivariable TAVI CPM for predicting 30-d mortality in UK-TAVI patients	(1) The final UK-TAVI CPM included 15 risk factors, which included 2 variables associated with frailty. (2) Scale demonstrated strong calibration and moderate discrimination

**Metze** **(2017)** [16]	N=213 Age 78 F 122 (42,7%)	PMVR 97 (45,5%)	(1) FFS	(1) procedural outcomes, short-term functional changes, and long-term clinical outcomes	(1) Mortality at 6 w was significantly higher in frail (8.3%) compared with nonfrail (1.7%) patients (p =0.03). Hazards of death (HR: 3.06; 95% CI: 1.54 to 6.07; p <0.001) and death or heart failure decompensation (HR 2.03; 95% CI 1.22 to 3.39; p. 0.007) were significantly increased in frail patients during long-term follow-up, which did not change relevantly after adjustment (2) PMVR can be performed with equal efficacy and is associated with at least similar short-term functional improvement in frail patients.

**Mok ** **(2016)** [61]	N=460; Age 81 ± 8 F 224 (49%)	TAVI Sarcopenia 293 (64%) Sarcopenic-Obesity 212 (46%)	(1) CT scan – SMM, FM	(1) assess the feasibility of evaluating body composition by CT (2) determine the prevalence of sarcopenia, obesity, sarcopenic obesity; (3) analyze the impact of differing body composition on 30-day and late clinical outcomes.	(1) Sarcopenia predicted cumulative mortality (HR 1.55, 95% confidence interval 1.02 to 2.36, p=0.04). (2) Differences in body composition had no impact on 30-day clinical outcomes after TAVI.

**Nemec ** **(2017)** [75]	N=238; Age 82.3 ± 10.0, F 78 (32,33%)	TAVI 53 (34%) sarcopenia,	(1) CT scan - SMI	(1) inter- and postprocedural complications (2) LOS, 30 d and 1-y mortality	(1) SMIs at L3 and T12 significantly correlated with prolonged LOS. (2) SMI3, SMI7 SMI12, SC, and BMI did not show a relationship with perioperative death or complications or 30- day and 1-year mortality rates. (3) VF showed a significant relationship with 30-day and 1-year mortality rates

**Okoh ** **(2017)** [34]	N=75; Age 92±2; F 49 (65%)	TAVI 30 (40%)	FFS	(1) all-cause mortality	(1) Significant improvement in overall health status of non-frail patients (mean difference: 11.03, P=0.032). (2) Unadjusted 30-day and 2-year mortality rates were higher in the frail group than the non-frail group (14% vs. 2% P = 0.059; 31% vs. 9% P = 0.018). (3) Kaplan-Meier estimated all-cause mortality to be significantly higher in the frail group (log-rank test; P = 0.042). (4) Frailty status was independently associated with increased mortality (hazard ratio: 1.84, 95% C.I: 1.06–3.17; P=0.028) after TAVI.

**Paknikar ** **(2016)** [76]	N=295; Age 74,68, F 102 (34,5%)	SAVR/TAVI NA	(1) CT scan - TPA	(1) To evaluate the use of sarcopenia as a frailty assessment tool	(1) 2 y survival was 85.7% in patients with sarcopenia, compared with 93.8% in patients without sarcopenia (P = .02). (2) Independent predictors of late survival included TPA (HR, 0.47; P = .02). (3) Male sex (OR, 0.52; P = .04) and TPA (OR, 0.6; P = .001) were predictive of high resource utilization. (4) A separate analysis by treatment group found that TPA predicted high resource utilization after SAVR (OR, 0.4; P<.001), but not after TAVI (P = .66)

**Puls** **(2014)** [77]	N=300; Age 82±5 years F 198 (66%)	TAVI 144 (48%)	(1) Katz Index	(1) all-cause mortality	(1) Early mortality was significantly higher in frail persons (5.5% vs. 1.3%, p=0.04 for immediate procedural mortality; 17% vs. 5.8%, p=0.002 for 30-day mortality; and 23% vs. 6.4%, p<0.0001 for procedural mortality). (2) In contrast, the Katz Index <6 was identified as a significant independent predictor of long-term all-cause mortality by multivariate analysis (HR 2.67 [95% CI: 1.7-4.3], p<0.0001).

**Rodés-Cabau ** **(2010)** [78]	N=339; Age 81±8; F 187 (55,2%)	TAVI 85 (25.1)	Eye ball test	(1) Procedural and 30-d outcomes	(1) Patients with either porcelain aorta (18%) or frailty (25%) exhibited acute outcomes similar to the rest of the study population

**Rodés-Cabau ** **(2012)** [79]	N=339; Age 81±8; F 187 (55,2%)	TAVI; 85 (25.1)	Eye ball test	(1) the occurrence of mortality (yes/no)	(1) At a mean follow-up 188 patients (55.5%) had died. (2) The predictors of late mortality were frailty (HR: 1.52, 95% CI: 1.07 to 2.17).

**Rodrigues ** **(2017)** [46]	N=221; Age 71, F 76 (34,3%)	Cardiac surgery; Pre-frailty 144 (65,15%)	(1) CSHA	(1) main outcomes after cardiovascular surgery in pre-frail patients compared with non-frail patients.	(1) Pre-frail patients showed a longer mechanical ventilation time (193 ± 37 vs. 29 ± 7 hours; p<0.05), LOS at ICU (5 ± 1 vs. 3 ± 1 days; p < 0.05) and total time of hospitalization (12 ± 5 vs. 9 ± 3 days; p < 0.05). (2) In addition, the pre-frail group had a higher number of AE with an increased risk for development stroke (OR: 2.139, 95% CI: 0.622–7.351, p = 0.001; HR: 2.763, 95%CI: 1.206–6.331, p = 0.0001) and in-hospital death (OR: 1.809, 95% CI: 1.286–2.546, p = 0.001; HR: 1.830, 95% CI: 1.476–2.269, p = 0.0001). (3) higher number of pre-frail patients required homecare services (46.5% vs. 0%; p < 0.05).

**Rodríguez-Pascual ** **(2016)** [35]	N=606; Age 83,95, F 355 (58%)	TAVI/SAVR 299 (49.3%)	(1) FFS	(1) all-cause mortality during the follow-up	(1) The HR (95% CI) of mortality among frail versus non-frail patients was 1.83 (1.33–2.51). (2) frailty criteria were considered separately; mortality was also higher among patients with slow gait speed [1.52 (1.05–2.19)] or low physical activity [1.35 (1.00–1.85)].

**Ryomoto ** **(2017)** [80]	N=85; Age 78 ± 6, F 57 (67%)	AVR 8 (9,4%)	(1) FIM	(1) whether the preoperative FIM is useful for decision making for a strategy in the era of TAVI	(1) The preoperative motor FIM score was significantly lower in the compromised group (45 ± 24) than in the unaffected group (85 ± 9, p =<0.01). (2) The duration of postoperative intubation, ICU stay, and postoperative hospitalization were significantly longer in the compromised group than in the unaffected group (48 ± 67 vs 16 ± 12 h, p<0.01; 6.7 ± 5.3 vs 3.4 ± 2.0 days, p<0.01; 34 ± 27 vs 23 ± 11 days, p = 0.02, respectively).

**Saji ** **(2016)** [81]	N=232; Age 80.1 ± 8.7 F 132 (57%)	TAVR; 77 (33,2%)	(1) CT scan - PMA (2) 5MWT	(1) all-cause mortality at 30 d and 6 mo.	(1) After adjustment for multiple confounding factors, the normalized PMA tertile was independently associated with mortality at 6 mo (adjusted HR 1.53, 95%, CI 1.06 to 2.21). (2) Kaplan-Meier analysis showed that tertile 3 had higher mortality rates than tertile 1 at 6 m (14% and 31%, respectively, p =0.029). (3) PMA is an independent predictor of mortality after TAVI; 5MWT combined with normalized PMA showed greater discrimination ability than alone.

**Saji ** **(2017)** [27]	N=155; Age 85 F 99 (65)	TAVI NA	(1) SPPB (2) MFFC (3) PARTNER scale (4) Frailty index (Japan) (5) CSHA (6) 5MWT	(1) all-cause unplanned readmission following TAVI	(1) Frailty markers other than MFFC were independently associated with unplanned readmission. (2) The analysis found that the SPPB, the PARTNER frailty scale, the frailty index, CSHA and 5MWT were independently associated with unplanned readmission even after adjustment in the multivariate analysis.

**Schoenenberger (2013)** [17]	N=119; Age 83.4+4.6 years; F 66 (55.5%)	TAVI 59 (49.6%)	(1) MGA/MGBE	(1) Functional decline over 6 mo	(1) The frailty index strongly predicted functional decline in univariable (OR per 1 point increase 1.57, 95% CI: 1.20–2.05, P = 0.001) and bivariable analyses (OR: 1.56, 95% CI: 1.20–2.04, P = 0.001 controlled for EuroSCORE; OR: 1.53, 95% CI: 1.17–2.02, P = 0.002 controlled for STS score). (2) Overall predictive performance was best for the frailty index [Nagelkerke's R2 (NR2) 0.135] and low for the EuroSCORE (NR2 0.015) and STS score (NR2 0.034). (3) In univariable analyses, all components of the frailty index contributed to the prediction of functional decline.

**Seiffert** **(2014) **[44]	N=845; Age 80.9 ± 6.5 F 432 (51.1%)	TAVI 16 (4.6)	(1) CSHA	(1) to determine the additional value of indicators of frailty for postoperative survival in the elderly patient sample in the last step	(1) BMI, eGFR, hemoglobin, pulmonary hypertension, mean transvalvular gradient and LV ejection fraction at baseline were most strongly associated with mortality and entered the risk prediction algorithm [C -statistic 0.66, 95 % confidence interval (CI) 0.61–0.70, calibration v2 -statistic = 6.51; P = 0.69]. (2) Frailty increased the C -statistic to 0.71, 95 % CI 0.65–0.76. (3) Frailty was strongly related to outcome and increased the discriminatory ability of the risk algorithm.

**Shimura** **(2017)** [47]	N=1215; Age 84.4 ± 5.0, F 854 (70,2%)	TAVI Moderately frail 122 (10.0%) Severely frail 48 (4.0%)	(1) CSHA (2) BMI (3) Albumin, (4) 5MWT (5) Hand grip.	The 30-d mortality and in-hospital mortality	(1) Cumulative 1-y mortality increased with increasing CSHA stage (7.2%, 8.6%. 15.7%, 16.9%, 44.1%, p<0.001). (2) In a Cox regression multivariate analysis, the CSHA (per 1 category increase) was an independent predictive factor of increased late cumulative mortality risk (HR: 1.28; 95% CI: 1.10-1.49; p<0.001).

**Stortecky ** **(2012)** [14]	N=100; Age 83.7± 4.6 F 60 (60.0%)	TAVI 49 (49.0%)	(1) MGA/MGBE	(1) outcomes at 30 day and 1 year	(1) Associations of cognitive impairment (odds ratio [OR]: 2.98, 95% confidence interval [CI]: 1.07 to 8.31), malnutrition (OR: 6.72, 95% CI: 2.04 to 22.17), mobility impairment (OR: 6.65, 95% CI: 2.15 to 20.52), limitations in basic ADL (OR: 3.63, 95% CI: 1.29 to 10.23), and frailty index (OR: 3.68, 95% CI: 1.21 to 11.19) with 1-year mortality were similar compared with STS score (OR: 5.47, 95% CI: 1.48 to 20.22) and EuroSCORE (OR: 4.02, 95% CI: 0.86 to 18.70). (2) Similar results were found for 30-day mortality and MACCE.

**Sunderman (2011)** [82]	N=400 Age 80.3 ±4 F 206 (51,5%)	Cardiac surgery Moderately frail 170 Severely frail 31	(1) CAF	(1) correlation of Frailty score to 30-d mortality.	(1) There were low-to-moderate albeit significant correlations of Frailty score with STS score and EuroSCORE ( p < 0.05). (2) There was also a significant correlation between Frailty score and observed 30-day mortality ( p < 0.05). (3) The comprehensive assessment of frailty is an additional tool to evaluate elderly patients adequately before cardiac surgical interventions.

**Sündermann ** **(2011)** [11]	N=400; Age 80.1 ± 4 years, F 206 (51,5%)	Cardiac surgery 114 (55,33% )	CAF	(1) 1 y all-cause mortality	(1) Patients who died within 1 y had a median frailty score of 16 [5;33] compared to 11 [3;33] to the 1 y survivors (P = 0.001). (2) 1 y mortality within each CAF subgroup shows a significantly higher mortality rate among patients in the “severely frail” compared to less frail groups. (3) Higher frailty group had a LOS at the ICU. (4) The CAF score facilitates prediction of mid-term outcome of high-risk elderly patients

**Yamamoto (2015)** [58]	N=777; Age 83,9, F 400 (51,48%)	TAVI 56 (7,2%)	BMI	(1) The 30- d mortality	(1) Kaplan-Meier curves indicated no significant differences in cumulative 30-d and 1-y survival. (2) BMI <20 was not associated with increased early or midterm mortality.

**Yamamoto (2017)** [58]	N=1215; Age 84.4 ± 5.0, F 854 (70.3%)	TAVI 284, (23,3%)	(1) Low albumin	(1) all-cause mortality after TAVI	(1) cumulative all-cause, cardiovascular, and noncardiovascular mortality rates were significantly higher in the low albumin group than in the normal albumin group (log-rank test, p <0.001, p = 0.0021, and p <0.001, respectively). (2) Poorer prognosis of the low albumin group in terms of cumulative all-cause and non-cardiovascular mortality was retained (p = 0.038, and p = 0.0068, respectively)

**Zuckerman (2017)** [83]	N=82; Age 71,6 F 24, 29%	Cardiac surgery NA	(1) CT scan - PMA	(1) postoperative LOS defined as the number of days from index procedure to hospital discharge	(1) Low PMA was correlated with lower handgrip strength and SPPB scores indicative of physical frailty. (2) Postoperative LOS correlated with PMA (R=L0.47, p =0.004), LMA (R=–0.41, p=0.01), and TMA (R= –0.29, p=0.03). (3) After adjustment PMA remained significantly associated with LOS (*β*= –2.35, 95% CI –4.48 to –0.22). (4) The combination of low PMA and handgrip strength, indicative of sarcopenia, yielded the greatest incremental value in predicting LOS.

*Intervention.* TAVI: Transcatheter Aortic Valve Implantation, SAVR: survival aortic valve replacement, PMWR: percutaneous mitral valve repair.

*Frailty Assessment.* FFS: Fried phenotype frailty index, SPPB: Short Physical Performance Battery, 5MWT: 5-meter walking test, CSHA: *Canadian Study of Health and Aging, *ISAR: Identification of Seniors at Risk, MMSA: MacArthur Study of Successful Aging, EMS: Elderly Mobility Scale, EFT: Essential Frailty Toolset, MFFC: Modified Fried Frailty Criteria, BMI: body mass index, SHERPA: Score Hospitalier d'Evaluation du Risque de Perte d'Autonomie, CGA: comprehensive geriatric assessment, MPI: Multidimensional Prognostic Index, UK-TAVI CPM: UK TAVI clinical prediction models, RAI: risk analysis index, SMI: skeletal muscle index, 6MWT: six-minute walking test, CAF: comprehensive assessment of frailty, PMA: psoas muscle area, SMM: skeletal muscle mass, FM: fat mass, TPA: total psoas area, LMA: lumbar muscle area, TMA: thoracic muscle area, VF: visceral fat, SC: subcutaneous tissue area, FIM: functional independence measure, MGA/MGBE: *Multidimensional Geriatric Assessment/*Modified Geriatric Baseline examination, *MMSE: *Mini Mental State Examination, *TUG: *Time Up and Go, *IDAL: *instrumental activities in daily living, FORECAST: Frailty predicts death One yeaR after Elective CArdiac Surgery Test.

*Outcomes.* AE: Adverse events, LOS: length of stay, STS: Society of Thoracic Surgery, QoL: quality of life, NYHA: the New York Heart Association, MACCE: major adverse cardiac and cerebrovascular events.

*Results.* OR: odds ratio, HR: hazard ratio, CI: confidence interval, NA: not available, ICU: intensive care unit, eGFR: estimated glomerular filtration ratio.

**Table 2:** Characteristics of reviewed studies on VHD and exercise training.

**Author** **(Year)**	**Study group Patient characteristics**	**CR variety** **CR length** **Intervention**	**Primary endpoint**	**Main results and adverse events**	**Conclusion**
Zanettini (2014) [84]	N=60; None; Age 83.5; Female 53%; TAVI CD - mentioned; Time to CR - 10,6 ± 3.4 d.	CR inpatient; Length 18.3 ± 5.6 days; Intervention: 6d/w (1) Intensity according to clinical condition, 6MWT, disability (2) In pts with mild disability interval or steady-state aerobic training and callisthenics was used.	(1) to determine the in-hospital and mid-term outcomes of these patients.	AE: NA 6MWT at discharge was significantly higher and corresponded to the 64% ± 23% of the predicted vs. 49% ± 21% in the admission test.	Most patients showed significant improvement in functional status, QoL, and autonomy, which remained stable in the majority of subjects during mid-term follow-up.

Russo (2014) [85]	N=158; TAVI = 78; SAVR = 80; Age 82.1; Female 60%; TAVI, SAVR; Time to CR - 13,7 ± 11.7 d	CR inpatient; Length 2 w; Intervention: 70% predicted max HR, RPE 3 sets of exercises, 6 d/w: 30 min of respiratory workout, aerobic session on a cycle and 30 min of callisthenic exercises. Aerobic session 10 min*⟶*30min	(1) The safety and efficacy of a structured, exercise-based CR program	AE: none All enhanced autonomy; 6MWT did not significantly differ (272.7±108 vs. 294.2±101 m, p. 0.42), neither peak-VO2 12.5±3.6 vs. 13.9± 2.7 ml/kg/min, p. 0.16.	CR is feasible, safe and effective in octogenarian patients after TAVI as well as after traditional surgery. CR rehabilitation programme enhances independence, mobility and functional capacity and should be highly encouraged

Pardaens (2014) [86]	N=145; VHD, risk, type of surgery; Age 64; Female 48,9%; AVR, MVR; CD - mentioned; Time to CR - 43±22 d	CR outpatient; Length 12-20 weeks; Intervention: 2-3/w for 60 min. Aerobic training, an intensity of the HR at AT combined with RPE. Strengthening exercises (15 minutes) at 60% of 1-RM for 2 sets of 15 to 20 repetitions.	(1) difference in exercise capacity early after VHD surgery (2) whether the functional improvement after training was affected by risk or type of surgery	AE: NA (1) Higher risk had a worse postoperative exercise capacity, higher VE/VCO2 (2) Exercise training - significant improvement in WL, peak VO2, AT, and 6MWD in each risk group (p <0.01). (3) Significant decrease in the VE/VCO2 in the medium- and high-risk patient groups (p <0.05).	(1) Exercise capacity after VHD surgery is related to the preoperative risk and to the type of surgery. (2) Similar benefit from exercise training can be obtained, independent of the preoperative risk class or the type of surgery. (3) ET should be offered to all patients after valvular surgery, regardless of their EuroSCORE risk or type of surgery

Fauchere (2014) [87]	N=112; TAVI vs SAVR; Age 79; Female 60%; TAVI, SAVR; CD - mentioned; Early phase CR.	CR inpatient; Length 3 weeks; Intervention: Training 2–3/d., 6 d./w. of supervised gymnastics, aerobic and respiratory workout sessions. Low/medium intensity (RPE).	(1) improvement during the CD in FIM-score, HADS-score and 6-MWT	AE: NA (1) 6-MWT in TAVI and SAVR patients (84.2 m ± 68.7 m vs. 82.8 m ± 65.1 m; p = 0.92) (2) total FIM score in TAVI and SAVR patients (9.9 ± 6.1 vs 12.2 ± 10.9; p=0.34).	(1) Patients in TAVI group were older and sicker than SAVR (2) Both patient groups did benefit in the same way from a post-acute in-patient rehabilitation program.

Baldasseroni (2016) [88]	N=160; Performance >15 vs. <15%; Age 80; Female 29,4%; CABG, HVS, CABG+HVS, ACS; CD - mentioned; Time to CR - 12 ± 10 d.	CR outpatient; Length 4 weeks; Intervention: 5d/w aerobic training program at an 60–70% of VO2 peak, RPE 11–13. Progressive increase in resistance on the basis of RPE, reevaluated weekly.	(1) effects of an exercise-based CR program on exercise tolerance and muscle strength (2) the independent predictors of changes in physical performance	AE: none Physical performance improved (VO2 peak, 10.9%; 6MWT, 11.0%; peak torque, 11.5%). higher baseline values predicting less improvement (VO2 peak: OR=0.86, 95% (CI)=0.77– 0.97; 6MWT: OR= 0.99, 95% CI=0.99–1.00; peak torque: OR=0.96, 95% CI=0.94–0.98).	(1) An exercise-based CR program was associated with improvement in all domains of physical performance even in older adults after an acute coronary event or cardiac surgical intervention, particularly in those with poorer baseline performance.

Voller (2015) [89]	N=442; TAVI = 76; SAVR = 366; Age 69.94; Female 38%; TAVI, SAVR; CD - mentioned; Early phase CR.	CR inpatient; Length 3 weeks; Intervention: 4-5/w. Aerobic exercise depending on the initial exercise intensity, outdoor walking, gymnastics and resistance training of the lower extremities	(1) the effect of CR on in patients after TAVI in comparison to patients after sAVR	AE: NA (1) 6-MWT and exercise capacity significantly increased in both groups (p<0,05). (2) After adjustment, changes were not significantly different between SAVR and TAVI, with the exception of 6-MWT (p. 0.004).	(1) Patients after TAVI benefit from CR despite their older age and comorbidities. (2) CR is a helpful tool to maintain independency for daily life activities and participation in socio-cultural life.

Savage (2015) [90]	N=576; HVS vs CABG vs CABG+HVS; Age 64.9; Female 22%; CABG, HVS; CD - mentioned.	CR outpatient; Length 12-20 weeks; Intervention: Aerobic training at 70- 85% of peak HR and/or RPE 12-14 for 45-60 min. Resistance training 1 set of 10 rep.	(1) If patients after HVD benefit similarly CR as CABG.	AE: NA (1) Peak VO2 increased 19.5% from 17.4±4.4 to 20.8±5.5 mL/O2/kg/min (p<0.0001). (2)	CABG and VHD patients experienced similar improvements in strength, and self-reported physical function and depression scores. Improvements in peak VO2 were similar between all groups

Pressler (2016) [91]	N=30; Intervention = 13; Control = 14; Age 81; Female 44%; TAVI; CD - mentioned; Time to CR 83 ± 34d	CR outpatient; Length 8 weeks; Intervention: 2-3/w 20min at 40%VO2 peak *⟶* 45 min at 70%VO2 peak by 8 week. (2) Resistance training in 2nd w 2/w at 30% 1-RM *⟶* 3 sets with 15 rep. at 50-60% 1-RM.	(1) difference in change in VO2 peak from baseline (2) Change in muscular strength, 6MWT, NYHA, QoL	AE: 3, not related (1) Significant changes in favor of Int. were observed for Peak VO2 (group difference, 3.7 mL/min per kg), muscular strength, components of QoL and 6MWT.	In patients after TAVI, ET appears safe and highly effective with respect to improvements in exercise capacity, muscular strength, and quality of life.

Sibilitz (2016) [92]	N=147; Intervention = 72; Control = 75; Age 62; Female 24%; Valve surgery; CD - mentioned; Time to CR - 1 mo	CR outpatient/home based; Length 12 w; Intervention: 3/w. (1) supervised training (69%) or (2) home-based training (31%). Intensity according to RPE increasing up to 12 w. The strength exercises; (2) cont. group not allowed to	(1) Improved physical capacity (VO2 peak) (2) Improved mental health.	AE: Int.:13pts vs cont. 3pts, not related (1) CR compared with cont. had a beneficial effect on VO2 peak at 4 months (24.8 mL/kg/min vs 22.5 mL/kg/min, p=0.045); (2) did not affect SF-36 MC at 6 mo (53.7 vs 55.2 points, p=0.40);	CR after HVD surgery significantly improves VO2 peak at 4 months but has no effect on mental health and other measures of exercise capacity and self-reported outcomes.

Genta (2017) [93]	N=135; TAVI vs SAVR; Age 80; Female 63%; TAVI, SAVR; CD - mentioned; Early phase CR.	CR inpatient; Length 4 w; Intervention: (1) 2/d. 30-min cycling/treadmill 6 d./w., starting at min*⟶*up to 14 RPE (2) OR 2/d. 30 min of walking along the 6MWT and pedal exerciser (0W) (3) 40-min 1/d respiratory exercise. (4) early mobilization	(1) Improved BI (2) Decreased risk of falls (3) Improved functional capacity	AE: 9pts (not related) (1) BI improved for all p<0,05 (from 73±23, to 90±16) (2) MFS decreased to all p<0,05 (from 30±21, to 25±17) (3) 6MWT improved for all p<0,05 (from 193 ±87, to 292±103)	(1) Intensive CR after TAVI is safe, well tolerated, and leads to a net improvement in disability, risk of falls, and exercise capacity, similar to that observed in less disabled SAVR patients.

Pollman (2017) [94]	N=168; None; Age 63; Female 16%; HVS, TAVI; CD - mentioned.	CR outpatient; Length 12 w; Intervention: 2/w 90 min and aerobic interval training at 60-80% VO2 peak; resistance training with 3 sets of 15 rep of 60% 1RM	(1) the effect of CR after VHD surgery on VO2 peak, long term morbidity, mortality	AE: none (1) VO2 peak improved by 16% from 21.6 to 24.8 mL/kg/min ( P < 0.0001) and 6MWT by 13% from 349 to 393 m (P = 0.0016).	CR after VHD surgery improved exercise capacity and was associated with reduced morbidity. Elderly were less likely to attend or complete CR and deserve special attention

Eichler (2017) [40]	N=136; None; Age 80.6; Female 52,5%; TAVI; CD - mentioned; Time to CR - 17.7±9,9 d.	CR inpatient; Length 3 Intervention: 5d/w 30 min the continuous/interval Patients (> 1.0 W/kg) strength training at 30–50% 1RM Outdoor walking, gymnastics, aqua gymnastics and spinal gymnastics in groups	(1) effect of a multicomponent inpatient CR after TAVI (2) predictors for the change in physical capacity, QoL.	AE: NA (1) 6MWD - 56.3 - 65.3m (2) max WL increased by 8.0 - 14.9 wat (p<0.001). (2) Higher cognition, nutrition and autonomy positively influenced the physical scale of SF-12. (3) baseline values of SF-12 had an inverse impact on the change during CR.	CR can improve functional capacity as well as QoL and reduce frailty in patients after TAVI.

*Intervention.* AVR: aortic valve replacement, MVR: mitral valve replacement, **PTMC: **Percutaneous Trans Mitral Comm**issurotomy**, TAVI: Transcatheter Aortic Valve Implantation, CABG: coronary artery bypass graft, HVS: heart valve surgery, ACS: acute coronary syndrome.

*Patients and Study Characteristics.* CR: cardiac rehabilitation, RPE: rate of perceived exertion, CD: comorbidities, HR: heart rate, 1-RM: 1 repetition maximum.

*Results.* AE: adverse events, WL: workload, CV: cardiovascular, AT: anaerobic threshold, QoL: quality of life, ET: exercise training, MFS: Morse fall scale, HR: hazard ratio, CI: confidence interval, OR: odds ratio, NYHA: New York Heart Association, 6MWT: six-minute walking test.
